# Myrtle berries seeds aqueous extract abrogates chronic alcohol consumption-induced erythrocytes osmotic stability disturbance, haematological and biochemical toxicity

**DOI:** 10.1186/s12944-018-0746-0

**Published:** 2018-04-23

**Authors:** Mohamed-Amine Jabri, Lamjed Marzouki, Hichem Sebai

**Affiliations:** Laboratoire de Physiologie Fonctionnelle et Valorisation des Bio-Ressources - Institut Supérieur de Biotechnologie de Béja, Université de Jendouba, Avenue Habib Bourguiba, B.P. 382 -9000 Béja, Tunisia

**Keywords:** Alcohol, Osmotic stability, Superoxide anion, Active compounds, Haematological parameters

## Abstract

**Background:**

This study examined the effects of chronic alcohol consumption in the rat erythrocytes membrane as well as the involvement of reactive oxygen species and proinflammatory cytokines in its pathogenicity in rats and evaluated the ameliorating effects of myrtle berries seeds aqueous extract (MBSAE).

**Methods:**

Fifty adult male *Wistar* rats were equally divided into five groups and treated daily for two months as follows: control, ethanol (3 g kg^− 1^ *b.w., p.o.*), and ethanol + MBSAE (25, 50 and 100 mg kg^− 1^, *b.w., p.o*.).

**Results:**

Exposure of rats to alcohol caused significant changes of some haematological parameters, enhanced erythrocytes hemolysis as well as an overproduction of reactive oxygen species such as H_2_O_2_, OH^•^ radical and superoxide anion, hence the increase of lipoperoxidation and the depletion of antioxidant enzymes activity as well as non-enzymatic antioxidant (-SH groups and GSH) levels. On the other hand, ethanol intoxication caused the increase of serum TNFα, IL-8, IL-6 and 1Lβ, markers of tissue inflammation. However, treatment with MBSAE alleviated all the deleterious effects of alcohol consumption.

**Conclusions:**

MBSAE possess active compounds, which exert marked protective effects in chronic alcohol intoxication, possibly by regulating the erythrocytes osmotic stability as well as antioxidant and inflammatory mediators.

## Background

The effects of alcohol on health are still a serious public health problem. Chronic alcohol intoxication is responsible for excess morbidity and mortality from cancer [1, 2], liver disease [3, 4], central or peripheral nervous system damage [5, 6], cardiovascular disease [7] or developmental abnormalities in children exposed in utero [8, 9]. Ethanol is a small molecule absorbed by simple diffusion. This diffusion is slow at the gastric level and the majority (70% to 80%) is absorbed in the small intestine (duodenum and jejunum) [10, 11]. The distribution of ethanol is very rapid (distribution half-life of 7 to 8 min) [12] to highly vascularized organs such as the brain, lungs and liver. The concentrations in these different organs are very quickly balanced with the blood concentrations [11]. Erythrocytes are the first line of cells affected by alcohol after the gastric mucosa cells. In fact, unmetabolised alcohol exerts direct effects, including hemolysis, and disruption of erythrocyte membranes by increasing fluidity and forming membrane pores [13, 14]. The production of reactive oxygen species seems to be a determining factor responsible for the erythrocytes membrane destruction observed in excessive alcohol consumers [15, 16]. Many studies have highlighted the production of free radicals during the metabolism of ethanol. These free radicals are at the origin of the lipoperoxidation of membranes and particularly the erythrocyte membranes [17–19]. The products of peroxidation are aldehydes such as malondialdehyde (MDA) and 4-hydroxy-2-nonenal (HNE). Unlike free radicals, these aldehydes are stable, diffuse in and out of the cell and can react with targets far from their formation site. They are not the final products of peroxidation, also act as “cytotoxic second messengers” and appear to be involved in most pathophysiological effects associated with oxidative stress [20].

On the other hand, hydrophilic and lipophilic compounds, which act by trapping or suppressing the generation of free radicals, are important in the control of intracellular redox homeostasis. In this context, myrtle (*Myrtus communis*) L. berries seeds can be used as an alternative treatment against alcohol toxicity and oxidative stress that is associated with him. Owing mainly to its richness in antioxidant and anti-inflammatory compounds such as polyunsaturated fatty acids [21] and phenolic compounds like flavonoids and anthocyanins [22] myrtle berries seeds extracts, are known to exhibit ROS scavenging activities [22], as well as protects against alcohol-induced gastric and duodenal damages [21].

These findings suggest that natural antioxidants may block the erythrocyte membranes destruction during alcohol exposure. Thus, the present study was designed with an aim to investigate the effect of myrtle berries seeds aqueous extract (MBSAE), a potent antioxidant against chronic alcohol-induced lysis of the erythrocyte membrane, haematological parameters disturbance and inflammation.

## Methods

### Chemicals

butylhydroxytoluene (BHT), trichloroacetic acid (TCA), 5,5-Dithio bis(2-nitrobenzoic acid) (DTNB), 2-Thio-barbituric acid (TBA), ethanol, ether, methanol, bovine serum albumin (BSA), bovine catalase, pyrogallol, beta-D-2-deoxyribose, Epinephrine, and NaCl were purchased from Sigma Chemical Co. (Sigma-Aldrich GmbH, Steinheim, Germany).

### Myrtle berry seeds aqueous extract preparation

Myrtle berry seeds were collected in November 2015 from the region of Nefza (North-West of Tunisia). The myrtle berry seeds were dried in an incubator at 50 °C for 72 h then ground in an electric mixer (MOULINEX Ovatio 2, FR). The seeds powder was then dissolved in distilled water and incubated at room temperature for 24 h under magnetic stirring. The sample was centrifuged at 10000 g for 10 min and the supernatant was lyophilized, aliquoted and stored at − 80 °C until use. Chemical composition of MBSAE (Table 1) was determined according to Jabri et al. [22].

**Table 1:** Characterisation of phenolic compounds of MBSAE by HPLC-DAD-ESI-MS/MS

compound no.	t_R_ (min)	λ max (nm)	[M-H]^−^ or [M-2H]^−^ (m/z)	Fragmention (m/z)	Tentative identification
1	6.71	254	299	137	Hydroxybenzoic acid hexose
2	7.94	276; 515	463	301	Delphinidin-3-*O*-galactoside
3	8.93	276; 520	463	301	Delphinidin-3-*O-*glucoside
4	9.04	354	463	301	Quercetin hexoside
5	10.33	276; 515	447	301	Delphinidin-3-*O*-rhamnoside
6	12.27	276; 515	609	301	Delphinidin rutinoside
7	15.95	276; 520	609	301	Delphinidin-3- (6 coumaroyl)-glucoside
8	17.25	278; 524	493	315	Petunidin-3-*O*- glucoside
9	20.38	278; 525	655	315	Petunidin diglucoside
10	29.25	278; 525	563	315	Petunidin malonylglucoside
11	30.66	525	621	315	Petunidin-3-*O*-rutinoside
12	30.74	278; 355	447	315	Isorhamnetin-*O*-pentoside
13	30.78	276; 516	615	299	Peonidin sambubioside
14	39.90	351	461	315	Isorhamnetin-*O*-rhamnoside
15	39.94	282; 527	511	331	Malvidin-*O-*galactoside
16	41.07	282; 527	511	331	Malvidin-*O*-glucoside
17	44.04	276; 516	641	299	Peonidin diglucoside
18	69.95	278; 525	461	331	Petunidin methyl pentose

### Animals and treatment

Healthy adult male *Wistar* rats (200–220 g body weight- 15 weeks old) were purchased from the Pasteur Institute of Tunis and used in accordance with the local ethics committee of Tunis University for the use and care of animals in accordance with the NIH recommendations [23]. They were provided with standard food (standard pellet diet- Badr Utique-TN) and water ad libitum and maintained in animal house at controlled temperature (22 ± 2 °C) with a 12 h light-dark cycle.

The animals were divided into five groups of 10 rats each. The first group served as control and received a physiological solution (NaCl, 0.9%, *p.o*.). The second group was given daily an oral administration of ethanol (3 g/kg, *b.w., p.o*.) prepared as a 35% (*v*/v) solution in 0.9% (*w*/*v*) NaCl. The groups 3, 4 and 5 was daily treated with ethanol (3 g/kg, *b.w., p.o*.) and MBASE (25, 50 and 100 mg kg^− 1^, *b.w., p.o*.) respectively. At the end of the 2 months treatment period, animals were fasted overnight and sacrificed. The blood sample was collected.

### Blood cells count and erythrocytes preparation

0.5 mL of blood sample was firstly collected in EDTA tubes for blood cells count using a haematology analyzer Coulter MAXM (Beckman Coulter, Inc., Fullerton, USA). And the rest was collected in heparinized tubes, whose a part is used to study the erythrocyte osmotic stability and tothe erythrocytes separation. Erythrocytes were isolated by gentle centrifugation (2000 g, 15 min at 4 °C), resuspended in isotonic phosphate buffer pH 7.4, and lysed with a hypotonic solution consisting of 20 mM Tris–HCl pH 7.2. Obtained homogenates were after used for biochemical determination of protein, ROS, SH-groups, reduced glutathione and antioxidant enzyme activities as well as MDA levels.

### Erythrocytes osmotic stability

To evaluate the effects of chronic EtOH consumption and MBSAE in a saline solution under physiological conditions on the erythrocytes behavior, the method described by Penha-Silva et al. [24] was used. Briefly, a set of Eppendorf flasks containing 1 mL of NaCl solution at concentrations ranging from 0 to 10 g/L were pre-incubated for 10 min at 37 °C.Then, 20 μl of whole blood ware added, the samples were afterwards vortexed, incubated for 20 min and centrifuged at 1300×g during 10 min. Absorbance at 540 nm was measured and converted to percentage hemolysis.

### Oxidative stress evaluation

MDA was estimated using the thiobarbituric acid test [25]. GSH levels were performed according to Sedlak and Lindsay method [26] and Thiol groups (-SH) were performed according to Ellman’s method [27]. SOD activity was measured according to the method described by Misra and Fridovich [28]. CAT activity was estimated using Aebi’s method [29]. GPx activity was determined according to the method described by Flohé and Günzler [30]. Finally, the protein content was determined according to Hartree [31] which is a slight change of the Lowry method.

### Measurement of ROS production

The intestine H_2_O_2_ level was performed according to Dingeon et al. [32]. Briefly, in the presence of peroxidase, the hydrogen peroxide reacts with p-hydroxybenzoic acid and 4-aminoantipyrine leading to a quantitative formation of a quinoneimine which has a pink color detected at 505 nm.

The hydroxyl radical level was determined using Payá et al. method [33]. Briefly, after oxidation of deoxyribose by hydroxyl radical generated by the Fe^3^ + −ascorbate-EDTA-H_2_O_2_ pathway and incubation with erythrocytes homogenate at 37 °C for one hour, the reaction mixture was stopped by adding of TCA (2.8%) and TBA (1%) and boiled at 100 °C for 20 min. Changes in absorbance were recorded at 532 nm against blank containing deoxyribose and buffer.

Superoxide radical was estimated according to Marklund and Marklund [34] with slight changes. Briefly, erythrocytes homogenates were incubated in Tris-HCl buffer, and then pyrogallol was added to the reaction mixture which will be incubated at 25 °C for fourminutes. The reaction has been stopped by HCl addition and absorbance was read at 420 nm against the blank.

### Assessment of cytokines: TNFα, IL-8, IL-6 and 1 L-1β in plasma

Cytokines (TNFα, IL-8, IL-6 and 1Lβ) levels were determined in the plasma samples using standard sandwich enzyme-linked immunosorbent assay (ELISA) kit according to the manufacturer’s instruction and expressed in pg per mg of proteins.

### Statistical analysis

All the data were expressed as mean ± standard error of the mean (S.E.M). Differences between the experimental groups were assessed by one-way ANOVA followed by Duncan’s test. Values were considered statistically significant when *p* < 0.05.

## Results

### Effect of chronic EtOH consumption and MBSAE on erythrocytes osmotic stability

**Fig. 1:**
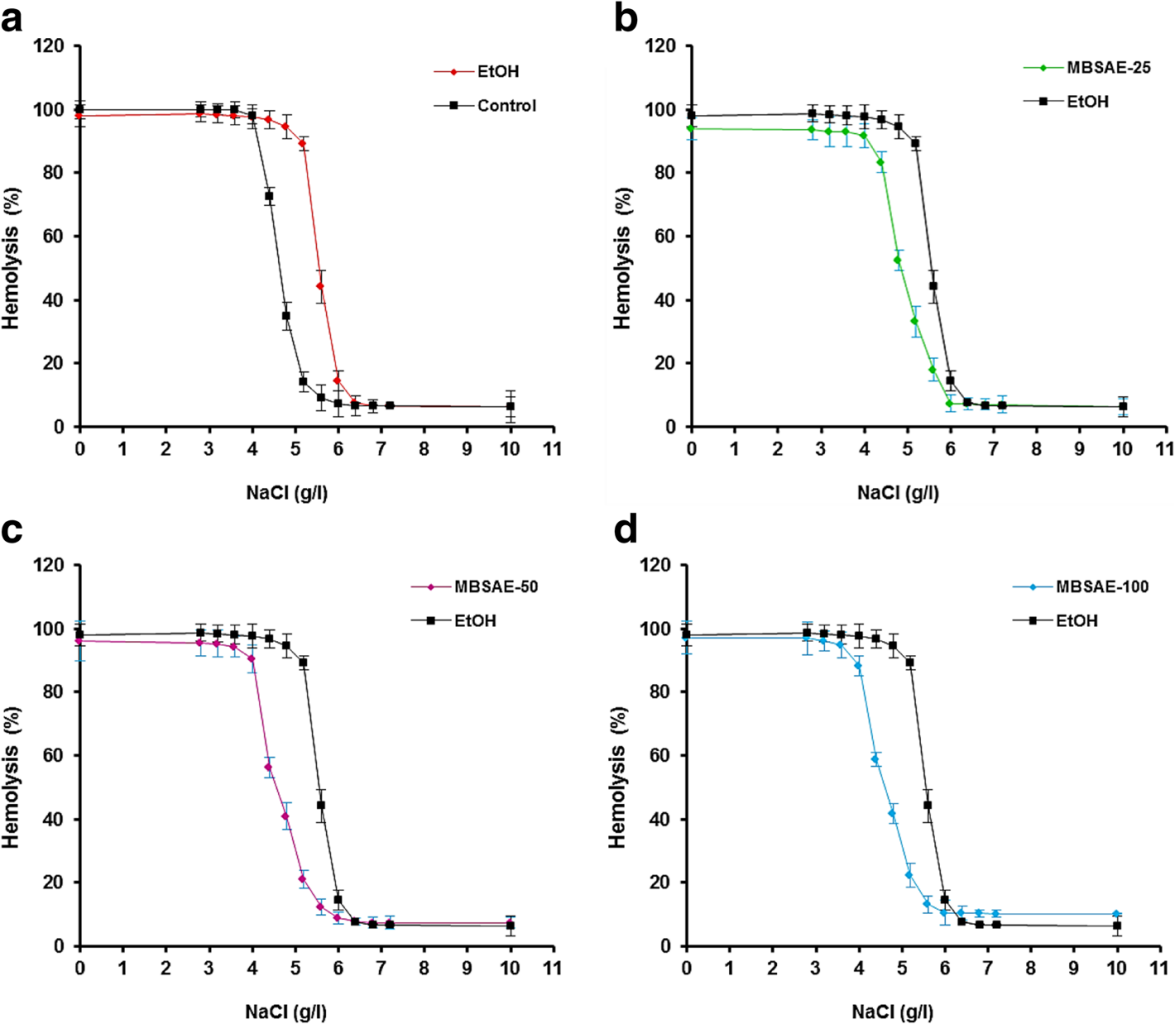
Effect of myrtle berries seeds aqueous extract (MBSAE) on chronic EtOH-induced disturbance in erythrocytes osmotic stability in physiological-like saline solution at 37 °C. **a** Osmotic stability curve of control rat erythrocytes compared to the ethanol (3 g kg-1 b.w., p.o./60 days) intoxicated groups. **b** Osmotic stability curve of MBSAE (25 mg kg-1, b.w., p.o.) treated rat erythrocytes compared to the ethanol (3 g/kg b.w/60 days) intoxicated groups. **c** Osmotic stability curve of MBSAE (50 mg kg-1, b.w., p.o.) treated rat erythrocytes compared to the ethanol (3 g/kg b.w/60 days) intoxicated groups. **d** Osmotic stability curve of MBSAE (100 mg kg-1, b.w., p.o.) treated rat erythrocytes compared to the ethanol (3 g/kg b.w/60 days) intoxicated groups. The data are expressed as mean ± S.E.M. (*n*=10)

As shown in Fig. 1, chronic ethanol intoxication caused a weakening of the erythrocyte resistance against hypotonic shock caused by different concentrations of sodium chloride compared to the control group as shown by the shift to the right of the hemolysis curve (Fig. 1a). However, MBSAE (25, 50 and 100 mg kg^− 1^, *b.w., p.o*.) significantly and dose-dependently increased the resistance of red blood cells, hence the inhibition of hemolytic activity caused by chronic consumption of ethanol and thus the inhibition of hemoglobin release.

### Effect of chronic EtOH consumption and MBSAE on haematological parameters changes

Table 2 shows the data of comparing haematological parameters. Alcohol consumption during two months caused a significant decrease of hemoglobin concentration (Hb), red blood cells number (RBC), mean corpuscular hemoglobin (MCH), hematocrit value (Ht), mean corpuscular hemoglobin concentration (MCHC) and platelets (Plt) number, on the other hand, a considerable increase in the mean corpuscular volume (MCV) level and white blood cells number (WBC) was marked. MBSAE treatment with the three increasing doses reversed the above changes of haematological parameters to near to baseline levels.

**Table 2:** Effect of myrtle berries seeds aqueous extract (MBSAE) on chronic EtOH-induced haematological parameters changes

Parameters	Experimental groups				
	Control	EtOH	EtOH + MBSAE-25	EtOH + MBSAE-50	EtOH + MBSAE-100
RBC count (10^6^/μl)	7.88 ± 0.76	6.47 ± 0.28	7.33 ± 0.41	7.47 ± 0.38	7.55 ± 0.77
Hb (g/dl)	14.63 ± 0.44	9.22 ± 0.21*	12.56 ± 0.32^#^	13.70 ± 0.88^#^	14.04 ± 0.90^#^
Ht (%)	47.58 ± 1.71	39.66 ± 1.44*	41.72 ± 1.14	43.58 ± 1.77^#^	45.36 ± 2.16^#^
MCV (mm^3^/RBC)	53.61 ± 2.43	68. 33 ± 2.71*	61.44 ± 3. 07	58. 33 ± 2.81^#^	55. 44 ± 2.09^#^
MCH (pg/RBC)	21.73 ± 1.51	19.39 ± 1.66	20.19 ± 2.39	20.08 ± 2.80	21.09 ± 2.26
MCHC (g/dl)	35.09 ± 1.12	30.22 ± 1.66*	32.14 ± 1.85	34.12 ± 2. 63^#^	34.80 ± 2. 47^#^
Plt (10^3^/μl)	741.70 ± 73.82	222.31 ± 72.88*	620.91 ± 66. 66^#^	681.15 ± 68.32^#^	711.06 ± 77.12^#^
WBC (10^3^/μl)	10.31 ± 0.45	17.21 ± 2.33*	13.51 ± 0.89^#^	12.77 ± 1.08^#^	10.81 ± 0.74^#^

Animals were daily treated with various doses of the MBSAE (25, 50 and 100 mg kg^− 1^, *b.w., p.o*.) or vehicle (NaCl 0.9%) before ethanol (30%, v/v, 10 mL kg^− 1^, b.w.) intoxication during two months. The data are expressed as mean ± S.E.M. (*n* = 10), *: *p* < 0.05 compared to control group and #: *p* < 0.05 compared to EtOH group

*RBC* Red blood cell, *MCV* mean corpuscular volume, *MCH* mean corpuscular hemoglobin, *Hb* Hemoglobin, *Ht* Hematocrit, *MCHC* mean corpuscular hemoglobin concentration, *Plt* Platelet and *WBC* white blood cells

### Effect of chronic EtOH consumption and MBSAE on erythrocytes lipid peroxidation

As expected, the EtOH group was characterized by a significant increase in erythrocytes MDA level, reflect of lipid peroxidation. While treatment with myrtle berries seeds aqueous extract (25, 50 and 100 mg kg^− 1^, *b.w., p.o*.) significantly decreased the erythrocytes lipid peroxidation (12.27 ± 1.44 nmol/mg protein for the high dose) compared to the EtOH control group (28.06 ± 2.59 nmol/mg protein) (Table 3).

**Table 3:** Effect of myrtle berries seeds aqueous extract (MBSAE) on chronic EtOH-induced erythrocytes oxidative stress

Treatment	MDA	SOD	CAT	GPx	-SH groups	GSH
Control	10.23 ± 1.12	37.93 ± 3.65	44.47 ± 4.42	26.48 ± 2.57	166.73 ± 9.84	44.84 ± 4.66
EtOH	28.06 ± 2.59*	22.16 ± 2.09*	26.28 ± 2.08*	14.31 ± 1.65*	73.66 ± 6.53*	19.69 ± 3.18*
EtOH + MBSAE-25	19.98 ± 1.31^#^	29.38 ± 2.17^#^	35.60 ± 2..56^#^	19.81 ± 1.42^#^	101.14 ± 6.33^#^	28.78 ± 4.11^#^
EtOH + MBSAE-50	14.47 ± 1.75^#^	32.60 ± 2.229^#^	38.73 ± 2.27^#^	21.37 ± 0.72^#^	122.47 ± 7.11^#^	33.47 ± 3.18^#^
EtOH + MBSAE-100	12.27 ± 1.44*	35.52 ± 2.83^#^	41.72 ± 3.58^#^	24.64 ± 1.81^#^	147.18 ± 8.41^#^	38.82 ± 3.64^#^

Animals were daily treated with various doses of the MBSAE (25, 50 and 100 mg kg^−1^, *b.w., p.o*.) or vehicle (NaCl 0.9%) before ethanol (30%, v/v, 10 mL kg^− 1^, b.w.) intoxication during two months

*MDA* nmol of MDA/mg protein, *SOD* units/mg protein, *CAT* μmol of H_2_O_2_ consumed/min/mg protein, *GPx* nmol GSH oxidized/min/mg protein, *-SH groups* μmol/mg protein, *GSH* nmol of GSH/mg protein

The data are expressed as mean ± S.E.M. (*n* = 10), *: *p* < 0.05 compared to control group and #: *p* < 0.05 compared to EtOH group

### Effect of chronic EtOH consumption and MBSAE on erythrocytes thiol groups and reduced glutathione

Erythrocytes thiol groups and reduced glutathione levels were decreased in EtOH-treated group as compared with respective controls. The animals treated with myrtle berries seeds aqueous extract exhibited significant and dose-dependant restoration in -SH groups and GSH (Table 3) levels.

### Effect of chronic EtOH consumption and MBSAE on erythrocytes antioxidant enzymes

The effect of aqueous extract of myrtle seeds on antioxidant enzymes activity in ethanol intoxicated rats is summarized in Table 3. Chronic alcohol consumption led to a significant decrease in the SOD, CAT and GPx activities in the erythrocytes as compared to the control group. While, treatment with MBSAE (25, 50 and 100 mg kg^− 1^, *b.w., p.o*.) significantly increased the antioxidant enzymes activity towards the normal value (*P* < 0.05) thus demonstrating the significant protective effect of MBSAE on chronic ethanol consumption-induced erythrocytes injuries.

### Effect of chronic EtOH consumption and MBSAE on erythrocytes ROS production

**Fig. 2:**
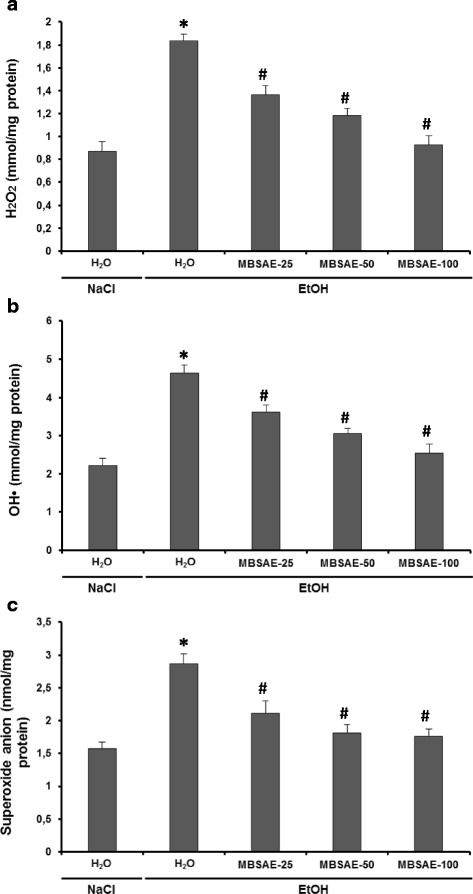
Effect of myrtle berries seeds aqueous extract (MBSAE) on chronic EtOH-induced disturbance in erythrocytes hydrogen peroxide (**a**), Hydroxyl radical (**b**) and superoxide anion (**c**). Animals were daily treated with various doses of the MBSAE (25, 50 and 100 mg kg^− 1^, *b.w., p.o*.) or vehicle (NaCl 0.9%) before ethanol (30%, *v*/v, 10 mL kg^− 1^, b.w.) intoxication during two months. The data are expressed as mean ± S.E.M. (*n* = 10), *: *p* < 0.05 compared to control group and #: p < 0.05 compared to EtOH group

The effects of the EtOH and MBSAE on erythrocytes reactive oxygen species were studied, and the results were summarized in Fig. 2. The administration of ethanol to rats during two months produced elevated erythrocytes oxidative stress, which was demonstrated by a significant increase in the hydrogen peroxide, hydroxyl radical and superoxide anion levels. MBSAE treatment showed protective effects against erythrocyte ROS overload induced by chronic ethane treatment, due to its ROS scavenging activities.

### Effect of chronic EtOH consumption and MBSAE on serum cytokines levels

**Fig. 3:**
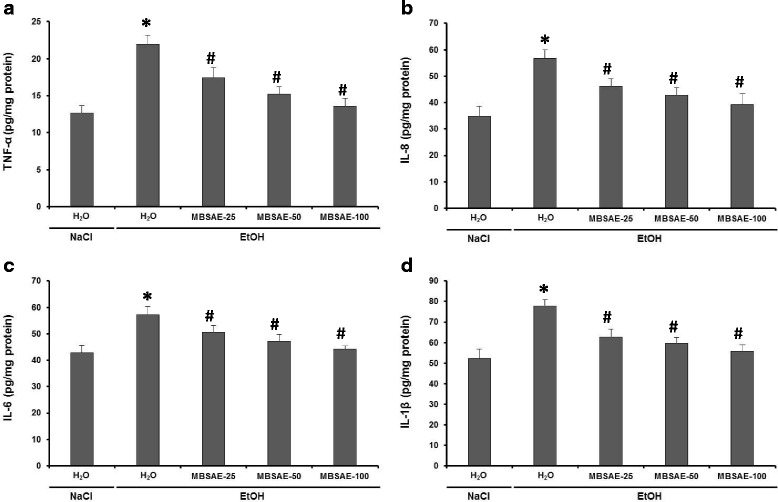
Effect of myrtle berries seeds aqueous extract (MBSAE) on chronic EtOH-induced disturbance in serum TNF-α (**a**), IL-8 (**b**), IL-6 (**c**) and IL-1β (**d**). Animals were daily treated with various doses of the MBSAE (25, 50 and 100 mg kg^− 1^, *b.w., p.o*.) or vehicle (NaCl 0.9%) before ethanol (30%, *v*/v, 10 mL kg^− 1^, b.w.) intoxication during two months. The data are expressed as mean ± S.E.M. (*n* = 10), *: *p* < 0.05 compared to control group and #: *p* < 0.05 compared to EtOH group

Enzyme-linked immunosorbent assay analysis was used to investigate the effects of EtOH and MBSAE (25, 50 and 100 mg kg^− 1^, *b.w., p.o*.) on serum TNFα, IL-8, IL-6 and 1Lβ levels (Fig. 3). Chronic alcohol consumption caused severe tissue inflammation, demonstrated by higher levels of plasma cytokines compared to the control. Nevertheless, MBSAE treatment significantly and dose dependently abolished the EtOH-induced inflammation.

## Discussion

The present study was undertaken to evaluate the effect of myrtle berries seeds aqueous extract against chronic ethanol consumption induces erythrocytes injuries and blood parameters changes.

We firstly showed that the exposure of rats to ethanol consumption during two months induced haematological changes by a decrease of Hb, RBC, MCH, Ht, MCHC and platelets number as well as increase in mean corpuscular volume level and white blood cells number. Indeed, the decrease of RBCs, Hb and Ht might be due to an inhibition of erythropoiesis and hemoglobin synthesis as well as to the increase in the level of erythrocytes destruction [35]. The increase in mean corpuscular volume and WBC number is related to the activation of defense and immune systems and showed that there were inflammations in the tissues [18]. The treatment with myrtle berries seeds aqueous extract returned these haematological parameters to near normal. Ethanol-induced blood parameters changes have been shown to be inhibited by many plant extracts such as chamomile [18] and *Opuntia ficus indica* juice [19].

On the other hand, we demonstrated that chronic ethanol intoxication acts on the osmotic stability of erythrocytes and causes their destruction by releasing their contents. In fact, the process of hemolysis is an irreversible phenomenon in which red blood cells are destroyed and release their content. Several factors are involved in hemolysis process citing, plasma, the anatomical state of the circulatory system and the functional status of the phagocytic mononuclear system [16, 36]. The haemolytic effect of a hypotonic solution is related to an excessive accumulation of fluid in the cell [37, 38] and attributed to its interaction with the sterols of the erythrocyte membrane which induces an increase of the membrane permeability and a movement of the ions: entry of Na^+^ and H_2_O, exit of K^+^, the membrane bursts, allowing the release of hemoglobin [39, 40]. Ethanol is also capable of promoting the lysis of erythrocytes, which has been attributed to the denaturing of membrane proteins [41]. However, an abundant literature is available concerning the involvement of ethanol in the lysis of the erythrocyte membrane [19, 24, 41].

Secondly, we showed in this study that MBSAE (25, 50 and 100 mg kg^− 1^, *b.w., p.o*.) effectively protected against erythrocyte lysis induced by chronic alcohol consumption and strengthens the resistance of red blood cells, thus testifying to a powerful anti-hemolytic activity. The lysis of the erythrocyte membrane has also been shown to be attenuated by many plant extracts such as *Salvia verbenaca* [42], *Allium rotundum* [43] and *Cyperus rotundus* [44] as well as by some pharmacological agents such as pyrrole carboxylic acids [45] and S-triazole-2-thiols [46].

In addition, taking alcohol for two months caused oxidative damage at the erythrocyte, manifested by the increase in the MDA level, an end product of lipid peroxidation and decreased the antioxidant enzyme activities such as SOD, CAT and GPx. Chronic alcoholism also induced a decrease of reduced glutathione (GSH) and sulphhydrils (-SH) groups levels. More importantly, myrtle seeds protect, significantly and dose dependently against all erythrocyte oxidative injuries induced by ethanol and return the values similar to those found in non-alcoholic animals at the highest dose. However, it is well established that induction of oxidative stress is part of the mechanisms of toxicity of alcohol [17]. If ethanol is not metabolized, it is able to trap the radical OH^•^; on the other hand, following its metabolism, it produces the reactive oxygen species (ROS) [47]. In addition, these ROSs contribute to its toxicity by triggering oxidative stress, but also accelerate its metabolism by the synthesis of the hydroxyethyl radical [48]. The particularity of ethanol-induced oxidative stress also lies in its ability to generate favorable conditions for the development of oxidative stress such as hypoxia, endotoxemia and cytokine release [17].

Erythrocytes are considered the major target of free radicals because their high polyunsaturated fatty acids (PUFAs) content, such as linoleic acid and arachidonic acid [49, 50]. In our study we showed that rats treated with ethanol showed marked higher erythrocytes ROS such as H_2_O_2_, hydroxyl radical and superoxide anion levels than all groups. Indeed, many studies have highlighted the production of ROS during the ethanol metabolism. These ROS are at the origin of the lipoperoxidation of the erythrocytes membranes. They may be derived from microsomal activity, mitochondrial respiratory chain, or oxidation of acetaldehyde by xanthine oxidase [51, 52]. In addition, the rate of hemolysis has been shown to be much higher when erythrocytes are treated with hydrogen peroxide [53]. This could be attributed to the oxidative nature of hydrogen peroxide and its ability to destroy the cell membrane and consequently the release of hemoglobin from cells. Hydrogen peroxide can also cause hydroxyl radical toxicity in provoking haem degradation of hemoglobin thus releasing Fe^2+^ ions which generates by the Fenton reaction the OH^•^ radical, more powerful thus contributing to lipid peroxidation [53, 54].

Moreover, we have shown that the antihemolitic activity of myrtle berries seeds aqueous extract is attributed in part to its ROS scavenging activity. Indeed, The MBSAE treatment has effectively inhibited the erythrocyte H_2_O_2_, OH^•^ radical and superoxide anion overload.

Otherwise, the HPLC-PDA-ESI-MS/MS analysis of MBSA revealed the presence 18 phenolic compounds, especially phenolic acids such as hydroxybenzoic acid hexose, flavonoids, like quercetin hexoside, isorhamnetin-*O*-pentoside and isorhamnetin-*O*-rhamnoside as well as anthocyanins, such as peonidin sambubioside, malvidin-*O*-galactoside, malvidin-*O*-glucoside, delphinidin-3-*O*-galactoside, delphinidin-*3-O*-glucoside, delphinidin-*3-O*-rhamnoside, delphinidin rutinoside, petunidin diglucoside, petunidin malonylglucoside, petunidin-*3-O*-rutinoside, delphinidin-3- (6 coumaroyl)-glucoside, peonidin diglucoside, petunidin-*3-O*- glucoside and petunidin methyl pentose [22, 55]. Indeed, these antioxidant molecules can reduce the toxicity of alcohol because they have a structure that allows them to trap free radicals by neutralizing them, which prevents ROS from reaching their biological targets [56]. In addition, the flavonoids that are identified in the MBSAE effectively inhibit have been shown to effectively inhibit the neutrophil myeloperoxidase (MPO), enzyme which use hydrogen peroxide (H_2_O_2_) to catalyze the production of the potent oxidants hypobromous acid (HOBr), hypothiocyanous acid (HOSCN) and hypochlorous acid (HOCl) [22]. Phenolic compounds identified in MBSAE has also inhibited the H_2_O_2_ and OH• radical produced by human neutrophils [22].

More importantly, our data showed also that MBSAE treatment abolished chronic EtOH-induced inflammation. Furthermore, clinical studies have shown that excessive alcohol users have higher levels of serum TNFα, interleukin-1 and interleukin-6 than controls [57, 58]. IL-8 is a chemotactic cytokine for neutrophils [59]. The main stimuli of IL-8 secretion are TNFα, IL-1 and endotoxin. Several studies have shown that TNFα, IL-8, IL-6 and 1Lβ are involved in the pathogenesis of alcoholic lesions of liver [60], brain [6] and stomach [61].

On the other hand, in a previous work we showed that MBSAE is very rich in polyunsaturated fatty acids (PUFAs), of which linoleic acid (80.78%) and oleic acid (6.34%) are the 2 major compounds [21]. in fact, polyunsaturated n-3 fatty acids exert its antiinflammatory effect by the competitive inhibition of eicosanoïd production from arachidonic acid. In addition, they inhibit the production and expression of proinflammatory cytokines, metalloproteinases [62]. In addition, omega-3 PUFAs intake reduces the proportion of arachidonic acid, resulting in decreased production of prostaglandin PGE-2. These PUFAs also inhibit the production of TNF-α and several interleukins by monocytes, macrophages or endothelial cells [63]. In this context, myrtle berries seeds aqueous extract (MBSAE) has been widely studied for their protective effect against duodenal [21], esophageal [64] and colon [22] inflammation.

In addition, several studies have shown that the chronic consumption of alcohol causes a significant fall of mitochondrial membrane potential [65, 66], which leads to cellular necrosis [67]. Moreover, the decrease in the potential of the mitochondrial membranes causes the opening of the ports of the transitions and permeability, these pores are generally opened by the ROS and cytokines [66]. In contrast, the ROS scavenging activity of MBSAE and its ability to inhibit proinflammatory cytokines restore the mitochondrial membrane potential and protect the cells against apoptosis and necrosis associated with the alteration of mitochondrial activities by alcohol consumption.

## Conclusion

In conclusion, alcohol consumption during two months induced overload of reactive oxygen species and the disturbance of erythrocytes redox balance leading to the haematological parameters change and lysis of the erythrocyte membrane as well as increased levels of TNFα, IL-8, IL-6 and 1Lβ, markers of tissue inflammation. But, the MBSAE offered significant protection against the chronic toxicity of EtOH by restoring these parameters to normal, which may be attributed to its chemical composition and their anti-inflammatory and ROS scavenging activities.

## Abbreviations

CAT: Catalase
EtOH: Ethanol
GPx: Glutathione peroxidase
GSH: Reduced glutathione
H2O2: Hydrogen peroxide
MBSAE: Myrtle berries seeds aqueous extract
MDA: Malondialdehyde
ROS: Reactive oxygen species
SH: Sulfhydril groups
SOD: Superoxide dismutase
